# Readiness and Challenges of E-Learning during the COVID-19 Pandemic Era: A Space Analysis in Peninsular Malaysia

**DOI:** 10.3390/ijerph20020905

**Published:** 2023-01-04

**Authors:** Adi Jafar, Ramli Dollah, Prabhat Mittal, Asmady Idris, Jong Eop Kim, Mohd Syariefudin Abdullah, Eko Prayitno Joko, Dayangku Norasyikin Awang Tejuddin, Nordin Sakke, Noor Syakirah Zakaria, Mohammad Tahir Mapa, Chong Vun Hung

**Affiliations:** 1 Faculty of Social Sciences and Humanities, Universiti Malaysia Sabah, Kota Kinabalu 88400, Sabah, Malaysia; 2 Department of Commerce & Management, Satyawati College (Eve), University of Delhi, New Delhi 110052, India; 3 Department of Copy Right Protection, Sangmyung University, 20, Hongimun 2-gil, Jongro-gu, Seoul 03016, Republic of Korea; 4 Faculty of Human Sciences, Sultan Idris Education University, Tanjong Malim 35900, Perak, Malaysia; 5 Faculty of Social Sciences and Humanities, Universiti Malaysia Sarawak, Kota Samarahan 94300, Sarawak, Malaysia

**Keywords:** online learning, online education system, university students, Geography Information Systems (GIS), cluster analysis

## Abstract

During the COVID-19 era, most countries, including Malaysia, have shifted from face-to-face teaching systems to online teaching programs. The aim of this study is to identify the main challenges that higher education students face during e-learning based on their residential location throughout Peninsular Malaysia. This study further examines the readiness of higher education students to apply e-learning. Therefore, a cross-sectional survey approach is used to fulfil the outlined objectives. Accordingly, 761 public (95.3%) and private (4.7%) higher education students residing in Peninsular Malaysia are sampled in this study. The survey was administered online for 37 days, from 21 October 21 to 6 December 2021, using either WhatsApp or Facebook. The raw data is inferentially (Principal Component Analysis, K-Means Clustering, Kruskal Wallis, and spatial analysis) and descriptively (mean, standard deviation & percentage) analyzed. It has been revealed that six clusters of students in Peninsular Malaysia face various challenges while following the e-learning program. Most states in Peninsular Malaysia are dominated by students in Cluster D (Terengganu, Perlis, Penang, Selangor, WP Kuala Lumpur, and WP Putrajaya) and Cluster B categories (Melaka, Johor, Kelantan, and Kedah). Students in the Cluster D category tend to suffer from physical health disorders and social isolation, while students in the Cluster B category face problems with decreased focus in learning, mental health disorders, and social isolation. The outcomes further indicate that the more challenges students face during e-learning programs, the lower their willingness to continue with the program. The results of this study are significant in addressing the challenges of e-learning, which will help stakeholders address and strengthen student abilities.

## 1. Introduction

In mid-December of 2019, the world’s population was shocked by the outbreak of the COVID-19 pandemic which occurred in Wuhan, Hubei, China [1,2,3,4]. The chain reaction of the pandemic contagion has led to changes in the education system [5], primarily in the teaching methods [6,7]. As a result, almost all countries, such as India, Chile, Ecuador, Italy, Mexico, Portugal, Poland, Romania, Turkey [8], China [9], and Malaysia [10,11,12], have shifted from using face-to-face teaching methods to online learning (e-learning), including at the tertiary level [8,13]. The ultimate purpose was to prevent the COVID-19 virus from spreading further [10,14]. It is undeniable that although implementing e-learning limits interactions, it simultaneously increases students’ flexibility and accessibility to participate in the learning process wherever they may be [13,15]. In fact, according to Moore and Diehl [16], e-learning has more advantages to offer than face-to-face learning since it is considered to be a more interactive, multi-modal, effective, convenient, ubiquitous form of learning, and is learner-centered.

However, the implementation of e-learning has also created challenges, especially for most developing countries. This is reasonable, since comprehensively applying e-learning is still novel for most developing countries, including Malaysia [6,10,17]. Most developing countries do not have adequate infrastructure and communications technology (ICT) to comprehensively support online teaching for all regions [17]. This is the case in Argentina, Brazil, and Chile, which face major internet connectivity and access issues [18]. Even countries like Bangladesh [19], Pakistan [20], and Mexico [21] are no exception in terms of facing similar problems. Most provinces in Lebanon also encountered infrastructure problems such as electricity and telecommunication deficits, which became the main hindrances to implementing e-learning [22].

A digital gap is another major challenge for implementing e-learning in several countries. In China, for instance, there is a digital education gap between students in urban and rural areas. Almost half of students in rural areas are unable to participate in e-learning due to limited electronic devices [23]. E-learning is even more difficult due to the low level of digital literacy among teachers and students who have less experience operating electronic devices and e-learning application tools [24,25,26]. The lack of readiness in participating in forced e-learning programs has led to mental health problems among students (depression, anxiety, and stress) [22,27,28,29,30]. Even the routine and regular practice of online teaching and learning can further lead to the risk of physical health problems such as lethargy, obesity, blurred vision, headaches, eye fatigue [31], and low sleep quality [32]. Several other studies, such as those by Abbasi et al. [33], John Lemay et al. [34], and Loganathan et al. [11], have also found that e-learning deprives students of interaction, thereby causing them to face social isolation. Ironically, the lack of social interaction in e-learning leads to a lack of motivation and feelings of loneliness and isolation [35].

Previous research indicates that students in developing countries still face numerous constraints and challenges when implementing e-learning, especially during the COVID-19 era. One developing country that is facing this challenge is Malaysia [10,11]. Until June 2022, the Malaysian government still implemented an online teaching system for all public and private universities. The low percentage of booster vaccine intake [36] and the outbreak of several COVID-19 variants (Delta, Omicron, and Deltacron) have become the main factors in the continuity of e-learning throughout Malaysia. Based on this situation, there is some question regarding the readiness of higher education students to continue following the e-learning teaching system.

Ironically, most previous research in Malaysia or globally only discusses the readiness and challenges faced by students when implementing e-learning, specifically in the context of a particular subject area or study program, such as the study of Chavarría-Bolaños et al. [37], Azlan et al. [38], Thapa et al. [6], Muthuprasad et al. [13], and Saiboon et al. [39]. The readiness and challenges of e-learning are rarely discussed based on the differences in the spatial aspects of the area. However, in developing countries (especially Malaysia), the location of a student’s residence is highly significant in influencing the challenges and readiness of e-learning. Therefore, this study identifies the main challenges that higher education students face in Peninsular Malaysia while implementing e-learning based on their residential location. In addition, this study examines the readiness of higher education students to apply e-learning.

## 2. Materials and Methods

### 2.1. Study Area

Peninsular Malaysia was chosen as the location for this study due to its highly dense population as compared to other regions, such as Sabah and Sarawak. Peninsular Malaysia consists of eleven states and two federal territories. The states and federal territories found in Peninsular Malaysia include Pahang (35,962 km^2^), Perak (21,022 km^2^), Johor (19,142 km^2^), Kelantan (15,105 km^2^), Terengganu (12,956 km^2^), Kedah (9471 km^2^), Selangor (8059 km^2^), Negeri Sembilan (6664 km^2^), Melaka (1655 km^2^), Penang (1040 km^2^), Perlis (813 km^2^), Federal Territory of Kuala Lumpur (243 km^2^), and Federal Territory of Putrajaya (49 km^2^) [40].

### 2.2. Data Collection

This research used a cross-sectional study design. A total of 761 higher education students in both public [19] and private [8] universities residing in Peninsular Malaysia were sampled in this study. According to Adam [41], to acquire continuous data with a minimum of 99% confidence level, only 463 respondents are required to represent a population size of more than 1,000,000 people. According to Isaac and Michael’s Table, the smallest sample size necessary to reflect an infinite population size or infinity (∞) is 349 individuals. In other words, regardless of the size of the study population, it may be adequately represented with a minimum sample size of 349 respondents [42]. This indicates that the sample number in this study is sufficient to represent the study population since it exceeds the minimum sample number required. The purposive sampling technique was employed to determine the number of respondents in this study. Only higher education students from public and private universities with active status were eligible to be included in the study sample. In order to prevent the possibility of COVID-19 transmission, the questionnaire was distributed online, through WhatsApp and Facebook using the KoBoToolbox software from 21 October to 6 December 2021. The e-questionnaire was circulated to academic WhatsApp groups (e.g., teaching course WhatsApp groups and student association WhatsApp groups) and university Facebook groups with the assistance of numerous university lecturers and students.

### 2.3. Questionnaire Design

The questionnaire consists of three main sections: Sections A, B, and C. Sections A and B involve a discussion of the demographic profiles of respondents (18 variables) and the challenges faced in implementing e-learning. Section C focuses on students’ readiness to participate in the learning system through e-learning, face-to-face, and hybrid methods. The types of questions in Sections B (35 variables) and C (3 variables) are similar since both use the Likert scale with five answer choices, ranging from 1 (strongly disagree) to 5 (strongly agree). The difference is that the questions in Section B are negative, while the questions in Section C are positive. The negative meaning in this case is that the higher the value of the Likert scale chosen (scale 5) the greater the amount of e-learning implementation issues experienced by students. A significant obstacle will undoubtedly have a negative effect on students. In contrast to Part C, the higher the Likert scale value (scale 5) the greater a student’s availability to execute one instructional method. In other words, a high Likert scale rating (scale 5) indicates good acceptance of a certain teaching strategy. Most research questions have been modified from the study of Adnan and Anwar [20], Zembylas et al. [43], and Kim et al. [44].

### 2.4. Validity and Reliability of the Instrument

Initially, pilot research was conducted to confirm the questionnaire’s validity and reliability. The minimal sample size for pilot research for testing reliability using Cronbach’s alpha is thirty respondents [45] or more [46]. Hence, the amount used in this pilot study was 50 sets. The analysis results found that the variables for constructs B and C are valid since they have a greater value of correlation coefficient (r_χγ_) than the critical value for Pearson’s correlation coefficient r [47]. This is because the minimum value of the correlation coefficient for the variables of construct B was 0.299, while for construct C, it was 0.397. On the other hand, the critical value for Pearson’s correlation coefficient r with a significance level of 0.5% was only 0.273 [48]. The reliability test did find that the alpha value for construct B was 0.935, while it was 0.6 for construct C. Cronbach’s alpha with a value of 0.935 is considered as excellent, while Cronbach’s alpha of 0.6 is satisfactory [49].

### 2.5. Data Analysis

The raw data was inferentially and descriptively analyzed using IBM SPSS statistics version 25. The inferential analysis included Principal Component Analysis (PCA), K-Means Clustering, the Kruskal Wallis test, and spatial analysis. Descriptive statistics consisted of the mean, standard deviation, and percentage.

**Figure 1:**
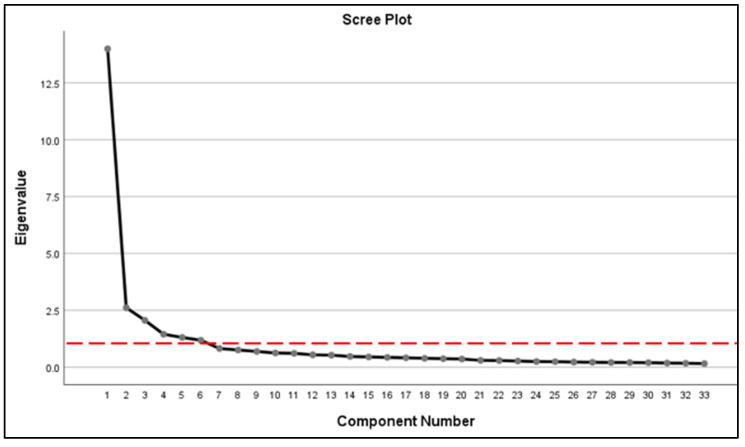
Number of major components.

The first stage of analysis is to summarize the data of construct B using the PCA analysis. This coincides with the function of PCA since it analyzes a data table from which several inter-correlated quantitative dependent variables describe observations. Its goal is to identify and extract key information from the statistical data to represent it as a set of new orthogonal variables called principal components (Mishra, 2017). The PCA analysis was conducted twice since the value of the loading factor (commonality) of the two variables (B1 & B19) were initially less than 0.5. Variable B1 refers to the group that suffers from Insomnia problems while variable B19 refers to the group that feels excessive task workload. Therefore, both variables were removed before beginning the PCA analysis a second time since only loading factor values of 0.5 are eligible to be re-analyzed [50,51]. The study outcomes of the Bartlett’s test were significant (x^2^ = 17775.55, df = 528, *p* < 0.05), indicating that the sample deserves further analysis. The number of factors was determined through the scree plot (Figure 1) for eigenvalues higher than 1 [52].

A total of six main components led to the cumulative value of variance of 68.5 percent (Table 1). This means that more than half (68.5%) of the challenges faced by higher education students in Malaysia are represented by these six components, while the rest are due to other factors [53]. For the humanities cluster, a cumulative value of variance of 50 percent is eligible for analysis [54,55].

The six main components were then analyzed using K-Means clustering. The goal is to group the samples into several clusters. This is accomplished by the analysis function of K-Means clustering which groups data into one group. The data in one group has different characteristics from the data in another group [56]. The following equation explains the K-Means clustering analysis:

$$J=\overset{k}{\underset{i=1}{\sum }}\overset{n}{\underset{j=1}{\sum }}{∥(x_{i}-v_{j})∥}^{2}=1$$

where, $∥x_{i}-v_{j}∥$ is the Euclidean distance between a point, $x_{i}$ And a centroid, $v_{j}$ iterated over all k points in the $i^{\mathrm{th}}$ cluster, for all n clusters.

**Figure 2:**
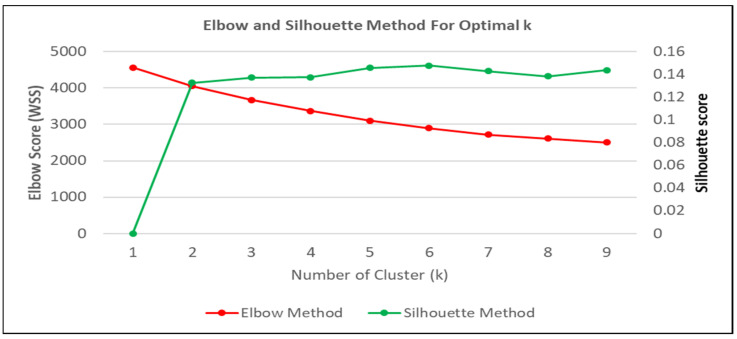
Optimal Number of Clusters.

The optimum number of clusters (k) is usually determined using the elbow and silhouette method [57,58,59]. Through the elbow and silhouette graph produced using machine learning analyses (Jupyter-Anaconda3), it was found that the optimal number of clusters in this study is six (Figure 2).

The next stage is to determine the mean values of the six components in each cluster. The purpose is to facilitate the interpretation of data on e-learning challenges based on the clusters. The mean values were grouped into three categories: low (1.00–2.33), moderate (2.34–3.67), and high (3.68–5.00) [60,61]. The next process is to map the cluster results according to state boundaries using GIS applications. The last step is to analyze Construct C using non-parametric analysis; i.e., a Kruskal Wallis test. It determines whether or not a significant difference is present between the six clusters in the readiness of e-learning, hybrid, and face-to-face methods.

### 2.6. Ethical Issues

All procedures have been carried out in accordance with the relevant regulations and guidelines. This study is being conducted in accordance with the Ethics Committee rules established by the Universiti Malaysia Sabah (UMS) Review Board (Ref No UMS/FSSK6.2/100-2/2/3). At the beginning of the online survey, all respondents in this study are obligated to read a consent letter before answering any questions. Furthermore, this study contained all important information, including its purpose and objectives. All participants are guaranteed their privacy, anonymity, and confidentiality.

## 3. Results

### 3.1. Demography

Of the total respondents in this study, 569 people (74.8%) were female, while the rest were male (192 people, 25.2%). There were more single students (750 people, 98.6%) than married students (11 people, 1.4%). Most respondents are studying in public universities (725 people, 95.3%) and consist of ethnic Malays (598 people, 78.6%) (Table 2).

### 3.2. Challenges of E-Learning on Higher Education Students in Peninsular Malaysia

During e-learning, tertiary students generally faced six major challenges (Table 3). The most dominant challenge is related to (Co1), decreased focus while learning, with a variance value of 20.26%. The next two challenges are (Co2), physical health disorders, and (Co3), basic amenities problems, with variance values of 12.12% and 10.82%, respectively. In addition, (Co4), mental health disorders (var (X) = 10.06%), and (Co5), not skilled in using e-learning (var (X) = 7.83%), are also issues commonly faced by students. The results of the PCA analysis further revealed that students face (Co6) social relationship problems during e-learning (var (X) = 7.41%).

### 3.3. E-Learning Challenges Based on Cluster

**Figure 3:**
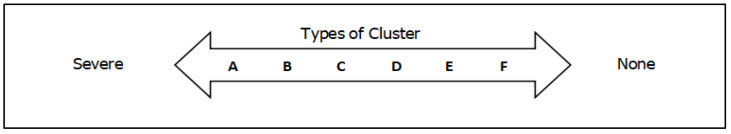
Comparison of severity levels between clusters.

A K-Means Clustering analysis shows that there are six clusters of higher education students in Peninsular Malaysia. The number of students in the Cluster B category is the largest (21%). Students in this category (Cluster B) must deal with the problems of decreased focus while learning (Co1) (M = 3.98, SD = 0.66), mental health problems (Co4) (M = 3.90, SD = 0.67), and social isolation (Co6) (M = 4.06, SD = 0.70). The students in the Cluster D category represents the second largest number of respondents (20.5%) in terms of dealing with physical health problems (Co2) (M = 3.90, SD = 0.58) and social isolation (Co6) (M = 4.09, SD = 0.61). Students in the Cluster C category (19.2%) tend to experience problems in terms of decreased focus while learning (Co1) (M = 3.86, SD = 0.61), physical health problems (Co2) (M = 3.83, SD = 0.58), and social isolation (Co6) (M = 4.03, SD = 0.67). The situation is different with Cluster E (19.1%). Students in that category (Cluster E) tend to experience physical health problems (Co2) (M = 3.97, SD = 0.63). The study also revealed that students in the Cluster F category (11.7%) are the least problematic (Table 4). The ranking of each cluster is shown in Figure 3. Based on the figure, Cluster A is the group of students with the most serious health problems. Cluster B came next, and then Cluster C. It was found that Cluster F students had the least problems compared to students in the other five clusters.

### 3.4. E-Learning Challenges Based on Location throughout Peninsular Malaysia

**Figure 4:**
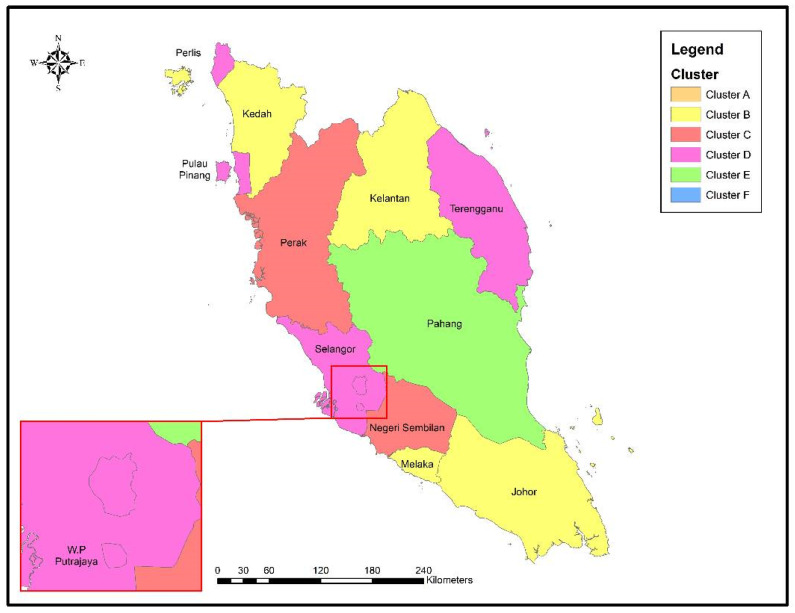
Types of clusters by states throughout Peninsular Malaysia.

The outcomes of the GIS mapping found that most states in Peninsular Malaysia, such as Terengganu, Perlis, Penang, Selangor, WP Kuala Lumpur, and WP Putrajaya, are dominated by the Cluster D category. On the other hand, the states of Melaka, Johor, Kelantan, and Kedah tend to be dominated by students in the Cluster B category. Students in the Cluster E category are mostly in one state, namely Pahang. In contrast, the other two states, Perak and Negeri Sembilan, are dominated by Cluster C (Figure 4).

### 3.5. Readiness of Students to Follow the E-Learning System, Hybrid, and Face-to-Face (F2F)

The average student is not prepared to return to the F2F learning method (M = 2.83, SD = 1.171). They prefer the hybrid (M = 3.19, SD = 0.922) or online (M = 2.99, SD = 1.133) approaches. The results of the Kruskal-Wallis test found that there are significant differences concerning the readiness of students to follow the e-learning system (*p* < 0.001) and F2F (*p* < 0.001). Students in Cluster F (MR = 517.83) and Cluster D (MR = 400.58), for instance, were found to be very willing to follow the online learning system. However, students from both groups (Cluster F: MR = 215.47, Cluster D: MR = 348.74) were not willing to follow the F2F learning system. In contrast, students in Cluster B (MR = 446.76) and Cluster C (MR = 424.46) are prepared to follow the F2F learning system. The students in the Cluster C category (MR = 354.14) are also not inclined to follow the online learning method (Table 5).

**Figure 5:**
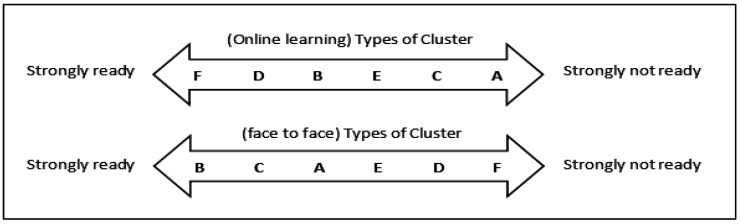
Students’ readiness to follow e-learning and face to face learning systems based on clusters.

If students were ranked by how likely they were to stay with the e-learning teaching system, Cluster F students would be the most likely to stay. Cluster D came next, then Cluster B.. The students in Cluster A are the least ready to use the e-learning teaching system. In contrast to the face-to-face system, the group of students in Cluster B has the highest level of availability, followed by Cluster C and Cluster A. The students in Cluster F are the least ready to follow the face-to-face teaching system. It can be said that students (clusters) who are ready for e-learning are not usually ready for face-to-face learning, and vice versa (Figure 5).

## 4. Discussion

During the COVID-19 age, the introduction of e-learning as an alternative to conventional teaching-learning strategies in the classroom is crucial. Without the use of this strategy during the lockdown in the majority of the countries, formal teaching-learning activities among students (from kindergarten to higher education) would certainly collapse [21,62].

On the one hand, it is believed that e-learning offers more benefits than face-to-face instruction owing to its greater interactivity, effectiveness, ubiquitous learning, multimodality, convenience and learner-centeredness [16]. Moreover, e-learning has been demonstrated to have a favourable impact owing to its greater feasibility, affordability, mobility, rich content and improved concentration [63].

However, this does not imply that the level of e-learning implementation and the challenges experienced by students are the same everywhere (district, state, and country). For instance, in the macro context, the issue of cost and access to the internet is not the primary challenge for developed countries [64], but it is a serious issue for the majority of developing countries, such as Ghana [65], Africa [66], Pakistan [67], and India [63]. This is typical given that the majority of developing countries have just recently implemented e-learning in all of their areas [6,10,17]. The problem of cost and access (Maheshwari, et. al. 2021) and the availability of digital devices [23], for example, is more prevalent in rural areas than in urban areas in the micro setting [63]. This indicates that, in the macro or micro context, there is a variation in the problems associated with the implementation of e-learning dependent on the location of residence.

In this study, higher education students experience six major hurdles while adopting e-learning: reduced attention while learning, physical health diseases, technical and connection issues, mental health disorders, inadequate digital literacy, and social isolation (Section 3.2). However, if viewed based on location, most states/regions in Peninsular Malaysia are dominated by students who have problems with social isolation (12 out of 13 states/federal territories) and physical health problems (9 out of 13 states/federal territories) (Section 3.4).

Although several previous studies on the challenges of e-learning in Malaysia have been conducted, such as the studies of Zulkifli et al. [68], Azlan et al. [38], and Moy and Ng [30], they have not addressed the issue of social isolation and physical health problems as a major form of e-learning challenge. The emphasis on the issue of social isolation, for example, is critical, as it can be a major factor in students dropping out of online courses or academic programs [69,70]. This is in line with the finding of Frankola [71], who discovered that the withdrawal rate (dropouts) for students who follow e-learning programs is higher (20–50%) than in face-to-face programs (10–20%). In Malaysia alone, during the COVID-19 pandemic (March 2020–July 2021), a total of 21,316 students dropped out. This is believed to be due to the implementation of teaching and learning from home [72].

The impact of e-learning on physical health disorders should also not be taken lightly. The number of students who suffer from this problem is significant, exceeding the percentage of students who suffer from mental health disorders (Section 3.3 and Section 3.4). Whereas in Malaysia, the mental health status of university students (depression, stress, & anxiety) during the COVID-19 pandemic belonged to the highly affected category [30]. The implementation of e-learning caused students to increasingly depend on electronic gadgets (i.e., computers, smartphones, and tablets). The period allotted to deal with electronic screen devices that emit various types of radiation is becoming longer as e-learning is conducted, causing headaches, itchy eyes, eye strain, and double or blurry vision [73]. The long-term use of electronic devices further increases the risk of muscle and bone problems such as shoulder and back pain [74] as well as neck pain [75]. As a result, several preventative measures must be implemented to mitigate these issues, such as blinking intentionally and regularly, avoid gazing at a specific location at all times, utilizing flicker-free displays, and performing mild workouts on specific areas of the body (hands, wrists, arms, neck, legs, & shoulders) [73].

It was further discovered that issues such as technology, connectivity, and low digital literacy, which are cited as major e-learning challenges in most developing countries [18,19,20,21,25,26], are not as serious in Malaysia. The evidence is that the mean values of the two challenges are the lowest compared to the other four challenges for all clusters (Section 3.3). No state/region throughout Peninsular Malaysia is dominated by students with technical and connectivity problems or low digital literacy (Section 3.4). This is not a surprise since the Malaysian Government has been actively committed to resolving technical and connectivity problems in former times (before the COVID-19 era). Numerous programs have been organized by the Malaysian government, together with several NGOs, to help low-income students (B40) improve their ability to adapt to the digital ecosystem. This includes the 1 Malaysia Netbook Free Giving Program [76], the Free Tablet Giving Subsidy Scheme [77], and the 2022 PerantiSiswa Program [78]. Through such programs, students are provided with electronic devices as well as free internet data plans for a year. From the 2022 PerantiSiswa program alone, it is estimated that 600,000 university students across the country will benefit [78]. This will certainly increase the ability of university students to follow e-learning programs more easily.

To further strengthen the implementation of e-learning programs in Malaysia and attract students to follow such programs, various aspects must be examined. This includes mitigation measures to overcome the challenges faced by university students while pursuing e-learning programs. Students’ readiness to follow e-learning programs is greatly influenced by the challenges they face throughout such programs. The more challenges or problems that students face, the lower their readiness to participate in these programs. As a result, this group (students who experience problems during e-learning) are more likely to choose the face to face teaching approach compared to e-learning, despite the pandemic (Figure 3 and Figure 5). To prepare students and ensure their well-being during e-learning, the target groups (Clusters A, B, & C), who mainly reside in the states of Melaka, Johor, Kelantan, Kedah, Perak, and Negeri Sembilan, must be immediately supported (Table 4 and Figure 4).

## 5. Limitations of the Study

This study has its own limitations, especially in terms of the data collection process. As explained in the methods section, this research survey was conducted online due to the COVID-19 pandemic. However, online surveys have the disadvantage of not getting responses from students who do not have internet access. In addition, the usage of online surveys leads to an imbalance in the number of samples acquired in each state. This is owing to the difficulties of collecting responses with the exact number of respondents in each state while conducting an online survey. In order to address this issue, the minimum sample size is exceeded to increase the total number of samples. For example, the minimum sample size required for this study is 463 respondents. However, the total sample size for this study was 761 people. In other words, 298 responders, or 64% of the required sample size, are overrepresented. This method is thought to be capable of alleviating the problem of sample quantity imbalance in each state. If this kind of study is to be undertaken again, it is suggested that a mixed method (online and face-to-face) be employed. This is because employing a mixed survey data collection strategy will make it easier to ensure that the number of samples obtained in each state is proportionate. Furthermore, obtaining samples in areas with restricted Internet access is simple.

## 6. Conclusions

It can be concluded that there are six clusters of students in Peninsular Malaysia who face challenges while following the e-learning program. Based on geographical location, most states in Peninsular Malaysia are dominated by students in Cluster D (Terengganu, Perlis, Penang, Selangor, WP Kuala Lumpur, and WP Putrajaya) and Cluster B (Melaka, Johor, Kelantan, and Kedah). Students in the Cluster B category were found to face more problems (decreased focus while learning, mental health problems, and social isolation) during the e-learning program than students in the Cluster D category (physical health problems and social isolation). Generally, the more challenges that students face, the lower their level of willingness to follow such programs. Students who experience numerous problems are more likely to follow face-to-face teaching than e-learning, even during a pandemic. Therefore, empowering students is necessary if authorities wish to continue implementing e-learning programs on an ongoing basis.

It is hoped that the study outcomes will help authorities (stakeholders/policymakers) in further strengthening the implementation of e-learning in Peninsular Malaysia. Empowering students would be easier and more methodical if specific knowledge is available regarding the difficulties or hurdles students experience depending on their residential area. The implications of the study outcomes can further contribute to the discoveries in the field of e-learning education. This study proved that the more problems or challenges students face during the e-learning program, the less likely they will be to continue the program. The research results indicate that in order to achieve success in adopting e-learning systems, the difficulties or obstacles experienced by students should not be overlooked, particularly when it comes to vulnerable student groups.

Compared to previous research, this study discussed the challenges of e-learning. There is no denying that several previous studies have discussed the challenges of e-learning. Nevertheless, the previous studies rarely focused on the challenges of e-learning in the context of geographical and spatial differences, particularly those involving mapping analysis. Therefore, this paper fills the previous research gap by understanding student behavior (challenges) using mapping analysis. The variables used in this study to understand the challenges of e-learning are also different from most previous research conducted in Malaysia. Previously, studies on the challenges of e-learning in Malaysia mostly revolved around the problem of decreased focus while learning, technical and connectivity issues, mental health problems, and low digital literacy. This study considered the four aspects as well as focused on social isolation and physical health problems which had not been previously highlighted.

## Figures and Tables

**Table 1:** Cumulative values of variance for the six main components.

Component	Initial Eigenvalues		
	Total	% Variance	Cumulative %
1	14.00	42.40	42.40
2	2.61	7.92	50.31
3	2.06	6.24	56.56
4	1.45	4.39	60.95
5	1.31	3.97	64.91
6	1.18	3.59	68.50
7–33	0.82–0.15	2.49–0.48	71.0–100

**Table 2:** Demographic Profile of Respondents.

Characteristics	Category	Frequency	Per Cent (%)
Gender	Male Female	192 569	25.2 74.8
Marital Status	Single Married	750 11	98.6 1.4
Ethnicity	Malay Chinese Indians Others	598 84 47 32	78.6 11 6.2 4.2
Institutional Status	Public university Private university	725 36	95.3 4.7

**Table 3:** Analysis Results of Main Component Extraction.

Component (Domain)/Item	Loading Factor	Variance (%)
Component 1 (Co1) Decreased Focus While Learning		
(B16) Lack of motivation since the learning environment at home is not similar to university	0.746	20.26
(B22) Easily bored due to very limited e-learning techniques	0.735	
(B21) Difficulty in focusing due to boring e-learning methods	0.734	
(B8) Lack of motivation due to not being able to meet friends and lecturers face to face	0.721	
(B18) Declining learning productivity	0.709	
(B17) Difficulty in understanding the content of the subject taught	0.656	
(B23) Difficulty focusing due to unconducive house condition	0.646	
(B20) Difficulty in completing group assignments digitally	0.613	
(B9) Feeling lonely	0.609	
(B15) Easily drowsy during classes	0.570	
(B24) Difficulty focusing due to disruption of other work at home	0.562	
Component 2 (Co2) Physical Health Problems		
(B3) Pain in the neck	0.778	12.12
(B6) Eye fatigue	0.770	
(B4) Pain in the back	0.766	
(B2) Headaches	0.662	
(B5) Blurred vision	0.661	
(B7) Fatigue	0.578	
Component 3 (Co3) Technical and Connectivity Problems		
(B29) My internet access is limited due to low internet network in my home area	0.812	10.82
(B28) My internet access is limited due to very expensive internet cost	0.806	
(B30) Power outages often occur at my house	0.738	
(B31) My personal laptop is slow	0.650	
(B32) I have to share a laptop with siblings	0.615	
Component 4 (Co4) Mental Health Problems		
(B12) Easily feel depressed (depression)	0.783	10.06
(B13) Easily experience stress	0.772	
(B14) Easily experience anxiety/restlessness (anxiety)	0.740	
(B10) Feel isolated	0.547	
(B11) Lack of personal/physical attention	0.533	
Component 5 (Co5) Low Digital Literacy		
(B35) Not easy to use e-learning like other systems	0.805	7.83
(B34) Not easy to become proficient in e-learning	0.777	
(B33) I found that e-learning is difficult to use	0.697	
Component 6 (Co6) Social Isolation		
(B25) Not close to peers	0.791	7.41
(B27) Unable to recognize many peers at university	0.754	
(B26) Difficulty communicating with peers online	0.737	

**Table 4:** E-Learning challenges based on cluster.

	Component (Co)	Co1	Co2	Co3	Co4	Co5	Co6	n	%
Cluster									
A	Mean (M)	4.19	3.87	2.56	3.73	1.96	4.12	65	8.5
	Standard Deviation (SD)	0.53	0.65	0.78	0.85	0.69	0.66		
B	Mean (M)	3.98	3.57	3.54	3.90	3.59	4.06	160	21
	Standard Deviation (SD)	0.66	0.76	0.73	0.67	0.77	0.70		
C	Mean (M)	3.86	3.83	3.16	2.80	3.48	4.03	146	19.2
	Standard Deviation (SD)	0.61	0.58	0.76	0.67	0.68	0.67		
D	Mean (M)	3.51	3.90	2.28	3.53	2.87	4.09	156	20.5
	Standard Deviation (SD)	0.69	0.58	0.72	0.71	0.70	0.61		
E	Mean (M)	3.67	3.96	2.79	3.42	3.03	2.83	145	19.1
	Standard Deviation (SD)	0.63	0.57	0.77	0.81	0.74	0.72		
F	Mean (M)	2.16	2.27	2.03	1.83	2.03	2.46	89	11.7
	Standard Deviation (SD)	0.66	0.71	0.76	0.59	0.82	0.86		

Mean value: 1.00–2.33 (Low) 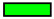, 2.34–3.67 (Moderate) 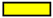, 3.68–5.00 (High) 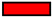.

**Table 5:** Kruskal-Wallis test results for e-learning, hybrid, and face-to-face learning methods.

Domain	Mean (M)	Standard Deviation (SD)	Types of Clusters	Mean Rank (MR)	*p*-Value
**E-learning**	2.99	1.133	A	293.22	<0.001
			B	364.23	
			C	354.14	
			D	400.58	
			E	360.85	
			F	517.83	
**Hybrid** **(Blended learning)**	3.19	0.922	A	396.27	0.992
			B	369.64	
			C	381.37	
			D	391.76	
			E	374.07	
			F	382.11	
**Face to face (F2F)**	2.83	1.171	A	402.18	<0.001
			B	446.76	
			C	424.46	
			D	348.74	
			E	391.49	
			F	215.47	

Kruskal-Wallis (*p*-value) at level of significance (α = 0.05).

## Data Availability

All data generated or analyzed during this study are included in this published article.
